# Identification of a Novel Imprinted Transcript in the Porcine *GNAS* Complex Locus Using Methylome and Transcriptome of Parthenogenetic Fetuses

**DOI:** 10.3390/genes11010096

**Published:** 2020-01-14

**Authors:** Jinsoo Ahn, Huiguang Wu, Joonbum Lee, In-Sul Hwang, Debing Yu, Jin-Seop Ahn, Jeong-Woong Lee, Seongsoo Hwang, Kichoon Lee

**Affiliations:** 1 Functional Genomics Laboratory, Department of Animal Sciences, The Ohio State University, Columbus, OH 43210, USA; ahn.134@osu.edu (J.A.); hgwu80@163.com (H.W.); lee.3920@buckeyemail.osu.edu (J.L.); yudebing@njau.edu.cn (D.Y.); 2 College of Veterinary Medicine, Yangzhou University, Yangzhou 225009, China; 3 Joint International Research Laboratory of Agriculture and Agri-Product Safety, the Ministry of Education of China, Yangzhou University, Yangzhou 225009, China; 4 Animal Biotechnology Division, National Institute of Animal Science, RDA, Jeonbuk 55365, Korea; insuri2642@korea.kr; 5 Department of Animal Breeding & Genetics, College of Animal Science & Technology, Nanjing Agricultural University, Nanjing 210095, China; 6 Biotherapeutics Translational Research Center, Korea Research Institute of Bioscience and Biotechnology, Daejeon 34141, Korea; ajsws@naver.com (J.-S.A.);

**Keywords:** genomic imprinting, *GNAS*, porcine, parthenogenetic, fetus, whole genome bisulfite sequencing, RNA-sequencing, differentially methylated region, parthenote

## Abstract

Genomic imprinting in domestic animals contributes to the variance of performance traits. However, research remains to be done on large-scale detection of epigenetic landscape of porcine imprinted loci including the *GNAS* complex locus. The purpose of this study was to generate porcine parthenogenetic fetuses and comprehensively identify imprinting patterns of the *GNAS* locus in transcript levels. To this end, both normally fertilized and bimaternal (uniparental) parthenogenetic porcine fetuses were generated, and whole genome bisulfite sequencing (WGBS) and RNA sequencing (RNA-seq) were performed to construct methylome and transcriptome, respectively. Differentially methylated regions (DMRs) between the fetuses were identified through methylome analysis, and parental-origin-specific expression patterns of transcripts were examined with transcriptome. As a result, three major DMRs were identified: paternally methylated *Nesp* DMR, maternally methylated *Nespas*-*Gnasxl* DMR, and maternally methylated *Exon1B–Exon1A* DMR. Parental-origin-specific expressions of those five DMR-affected transcripts were found, including a novel imprinted transcript, *Exon1B*, in pigs. In conclusion, using parthenotes, parental-origin-specific imprinting patterns in the porcine *GNAS* locus was comprehensively identified, and our approach paves the way for the discovery of novel imprinted genes and loci in a genomic context across species.

## 1. Introduction

Genomic imprinting in mammals occurs in a subset of genes, often in clusters, and results in epigenetic regulation of monoallelical gene expression in a parental-origin-specific manner [1]. Imprinting has been extensively studied in mice and humans to understand epigenetic architecture of complex diseases and traits [2,3,4,5,6,7]. For example, allele-specific deficiency of imprinted guanine nucleotide-binding protein, α-stimulating (*GNAS*) gene products led to various metabolic phenotypes: obesity and hypometabolic states (in maternal knockout of Gsα, or *Gnas*) and leanness with increased metabolic rate (in paternal knockout of XLαs, or *Gnasxl*) [5,6,7]. Studies on domestic animals have further reported the contribution of genomic imprinting to the variance in performance traits [8,9]. Considering the economic importance of those traits and the possible translation of animal studies to human biology, comprehensive examination of imprinted genes and loci is of great interest to both animal and biomedical industries. However, previous approaches of identifying imprinted genes in animals based on single nucleotide polymorphisms (SNPs) or sequence deletions/insertions might face efficiency limitation when there is a lack of informative variations. To overcome this issue, the generation of uniparental fetuses followed by genome-wide detection of epigenetic landscape may be a definitive approach to pursue, especially for complex loci such as the locus of *GNAS*. The *GNAS* complex locus has a complicated imprinting pattern due to alternative promoter usage and extensive differentially methylated regions (DMRs) on the promoters [10]. Among multiple groups of transcripts, the maternal expression pattern of *Nesp* in the pig has been reported [11]. Nevertheless, comprehensive expression patterns of multiple groups of transcripts on the porcine *GNAS* locus remain to be elucidated. 

By comparing parthenogenetic (PA) and control (CN) fetuses, we performed whole genome bisulfite sequencing (WGBS) for methylome profiling of the imprinting control region (ICR) in the *GNAS* locus and conducted RNA sequencing (RNA-Seq) for comprehensive transcriptome analysis. Our results revealed the full-scale imprinting status of the *GNAS* locus, including the discovery of a novel imprinted transcript, and confirmed the appropriateness of the strategy to identifying imprinting patterns using WGBS and RNA-Seq of parthenogenetic fetuses. 

## 2. Materials and Methods 

### 2.1. Ethics Statement

Our study protocol and standard operating procedures for the treatment of the pigs used in this study were reviewed and approved by the Institutional Animal Care and Use Committee of the National Institute of Animal Science, Rural Development Administration of Korea (approval number NIAS2015-670).

### 2.2. Porcine Oocyte Collection and In Vitro Maturation

Pig oocyte collection and in vitro maturation (IVM) were performed following previously described procedures [12]. Briefly, porcine ovaries were obtained from a local slaughterhouse (Nonghyup Moguchon, Gimje, Korea), immediately transported to a laboratory in normal saline solution supplemented with penicillin, and maintained in a thermos at 30–35 °C. Cumulus-oocyte complexes (COCs) were collected and washed in Tyrode’s lactate-Hepes containing 0.1% (w/v) polyvinyl alcohol. Oocytes with several layers of cumulus cells were selected and washed three times in TCM-199 (GIBCO, Grand Island, NY, USA) supplemented with 0.1% polyvinyl alcohol (w/v), 3.05 mM D-glucose, 0.91 mM sodium pyruvate, 0.57 mM cysteine, 0.5 µg/mL luteinizing hormone, 0.5 µg/mL follicle stimulating hormone, 10 ng/mL epidermal growth factor, 10% porcine follicular fluid (pFF), 75 µg/mL penicillin G, and 50 µg/mL streptomycin. For IVM, 50 COCs were transferred into 500 µL of maturation medium in a four-well dish (Nunc, Roskilde, Denmark). The oocytes were matured for 40–42 h at 38.5 °C in an incubator with 5% CO_2_.

### 2.3. Production of Parthenogenetic Embryos

After 40–42 h of IVM, cumulus cells were removed and oocytes with the first polar body were selected for activation. Those chosen oocytes were placed into 250 µm diameter wire electrodes of a fusion chamber (BLS Fusion = Eelctrode, Budapest, Hungary) covered with 0.3 M mannitol solution containing 0.1 mM MgSO_4_, 1.0 mM CaCl_2_, and 0.5 mM Hepes. For fusion, two DC pulses (1 s interval) of 1.2 kV/cm were applied for 30 µs using an LF101 Electro Cell Fusion Generator (Nepa Gene Co., Ltd., Chiba, Japan). After electric stimulation, 200 parthenogenetic embryos were transferred into both oviducts of each of the two LY (Landrace X Yorkshire) surrogate gilts aged 12 months at onset of estrus to produce parthenogenetic fetuses. 

### 2.4. Collection of Fertilized and Parthenogenetic Fetuses

Two LY gilts were mated with boars to provide fertilized control fetuses for this study. Gilts were naturally mated twice with a 6 h interval during their natural heat (onset of estrus = day 0). Considering that the monoallelic expression of some imprinted genes does not occur until after the blastocyst stage [13] and porcine parthenogenetic conceptus dies at approximately day 32 of gestation [14], both normally fertilized and parthenogenetic fetuses were recovered at day 21 from the gilts and surrogates, respectively, that had been confirmed pregnant by an ultrasound examination. For the recovery of fetuses, the gilts were euthanized and their reproductive tracts were dissected. Fetuses surrounded by placenta were then gently separated from the uterus using two pairs of forceps. Subsequently, fetuses were removed from the placenta and placed on a piece of clean, dry tissue using a pair of forceps in order to dry liquid on the surface of the fetus. Only fetuses with intact morphology and comparable sizes were selected. Lengths of fetuses were 2.2, 2.1, and 2.1 cm for normal control (CN) and 2.0, 2.0, and 1.6 cm for parthenotes (PA). The collected fetuses were stored in liquid nitrogen until further use. 

### 2.5. Whole Genome Bisulfite Sequencing (WGBS)

Pig genomic DNA was isolated from the whole collected fetuses of CN and PA (n = 3 for each) and fragmented. Accel-NGS Methyl-Seq DNA Library Kit (Swift Biosciences, Inc. Ann Arbor, MI, USA) was used for optimized bisulfite conversion of genomic DNA according to the manufacturer`s instructions. To amplify the bisulfite-treated DNA, PCR was conducted with adapter primers and Diastar™ EF-Taq DNA polymerase (Solgent, Daejeon, Korea). The thermal PCR conditions are: 3 min at 95 °C followed by 35 cycles of 30 s at 95 °C, 30 s at 60 °C, and 30 s at 72 °C, and a final 5 min at 72 °C. The PCR products were subjected to bead-based clean-up and then sequenced using HiSeqX sequencer by Macrogen (Seoul, Korea). 

### 2.6. Analysis of WGBS

Total raw bases ranging from 127.10 to 131.13 Gbp were generated for each of the samples (3 CN and 3 PA groups). After data filtering and trimming using Trim Galore (v0.4.5), clean reads (ranged 839.71–866.48 Mbp) for each sample were left. Those reads were mapped to the reference genome (Sscrofa11.1/susScr11) with BSMAP (v2.87) and uniquely mapped reads were selected to sort and index. The methylation ratio of every single cytosine location was extracted from the mapping results using ‘methylatio.py’ script in BSMAP (Supplementary Table S1). Analysis of DMRs was performed using the program metilene (v0.2-8) [15] on the methylation ratios at all CpGs to identify DMRs with >10 CpGs, a genomic distance between CpGs < 300 bp, and a mean methylation difference between groups of >0.2. Significant DMRs with false discovery rate (FDR) < 0.05 were retained (Supplementary Table S2). The R/Bioconductor package Gviz (v1.28.3) [16] were used to visualize the methylation ratios and significant DMRs in relation to genomic coordinates of the *GNAS* locus.

### 2.7. RNA Extraction from the Fetuses

Total RNA from whole CN fetuses (n = 3) and whole PA fetuses (n = 3) samples was isolated with TRIzol reagent (Sigma-Aldrich, USA) according to the manufacturer’s instructions. The RNA samples were first treated with DNase I to degrade any possible DNA contamination. The RNA integrity was evaluated by 1.2% agarose gel electrophoresis and calculated by the ratio of 28S/18S rRNA (>2) and the RNA integrity number (RIN) (RIN > 7) using an Agilent 2100 BioAnalyzer. The RNA concentrations were assessed by the ratios of A260/A280 and A260/A230 (1.8–2.0).

### 2.8. cDNA Library Construction and RNA-Sequencing

In order to construct cDNA libraries with the TruSeq RNA Sample Prep Kit v.2 (Illumina, San Diego, CA, USA), 1ug of total RNA was used. Using the protocol consisted of polyA-selected RNA extraction, RNA fragmentation, random hexamer primed reverse transcription and amplification, the final cDNA library was generated. The libraries were quantified using quantitative Real-Time PCR (qPCR) according to the qPCR Quantification Protocol Guide and qualified using an Agilent Technologies 2100 Bioanalyzer. The library products (100nt paired-end) were sequenced by Illumina HiSeq2500. 

### 2.9. De novo Assembly of Sequencing Reads

The raw sequencing reads were filtered, and the reference genome sequence of *Sus scrofa* (Sscrofa11.1/susScr11) and annotation files were downloaded from the Assembly database at the National Center for Biotechnology Information (NCBI) (http://www.ncbi.nlm.nih.gov/assembly/). The clean sequencing reads were then aligned to the reference genome using HISAT2 (v2.1.0) [17] with default parameter settings, except for the parameter—dta for tailored transcriptome assembly. RNA-Seq alignments in BAM format were generated and sorted by SAMtools (v1.9), and then the read coverage extracted from BAM files was plotted in alignment tracks and visualized using the R/Bioconductor package Gviz (v1.28.3) [16]. 

### 2.10. Analysis of Differential Expression of Exonic Regions

Using the R/Bioconductor package DEXSeq (v1.30.0) [18], RNA-seq reads were counted and analyzed. To prepare non-overlapping exon counting bins in GFF format, the Python script ‘dexseq_prepare_annotation.py’ from the DEXSeq package and the annotation file were used. Then, the ‘dexseq_count.py’ script was used to count reads at each counting bin using each BAM file as an input and generate count tables. The count tables and GFF file were used as the input files in DEXSeq for differential analysis. The counts from individual fetal samples were normalized for different sequencing depth by estimated size factors [18]. Exonic regions that are differentially expressed between CN and PA, also termed differential exon usage (DEU) [18], were identified with default settings and the function testForDEU of DEXSeq at a FDR of 0.05 (Supplementary Table S3). The term “E” was used to denote an exon counting bin (e.g., E001 for *Nesp* 1st exon).

## 3. Results

### 3.1. DNA Methylation in the GNAS Locus of Porcine Parthenogenetic Fetuses is Regulated in an Exon- Specific Manner 

**Figure 1:**
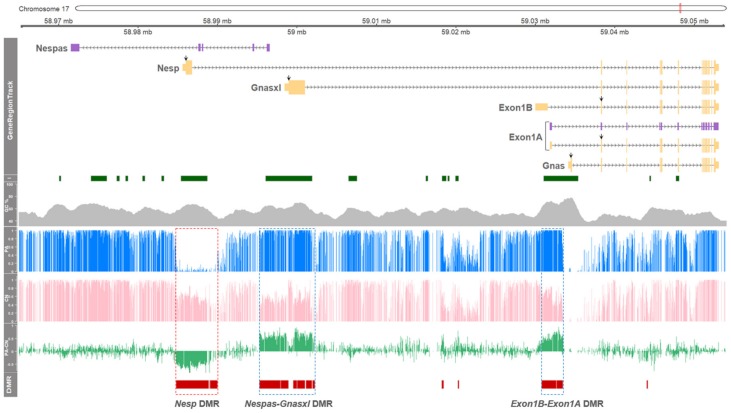
DNA methylation profiles of the *GNAS* locus in porcine parthenotes determined by WGBS. The 81.5-kilobase-pair region of *GNAS* locus on porcine chromosome 17 (chr17:58,971,522 – 59,053,022) is schematically represented in GeneRegionTrack. Six transcripts are indicated by yellow boxes for protein-coding transcripts (tall boxes: coding region; short boxes: noncoding region; black downward arrows: ATG start codon) or by purple boxes for noncoding transcripts, and directions are marked by black horizontal arrows. The transcript information including protein-coding and noncoding was based on the NCBI Gene and Nucleotide databases (https://www.ncbi.nlm.nih.gov/ gene and https://www.ncbi.nlm.nih.gov/nuccore). From I track to DMR track: CpG islands (I; dark green boxes), GC content (GC %; grey area), mean methylation ratios of parthenotes (PA, blue histogram lines), mean methylation ratios of controls (CN, pink histogram lines), mean methylation differences between PA and AI (PA-AI, green histogram lines), and significant DMRs (DMR, red horizontal bars; FDR < 0.05) are illustrated. A hypomethylated DMR in PA is indicated by a dotted red box, and hypermethylated DMRs in PA are indicated by dotted blue boxes.

**Figure 2:**
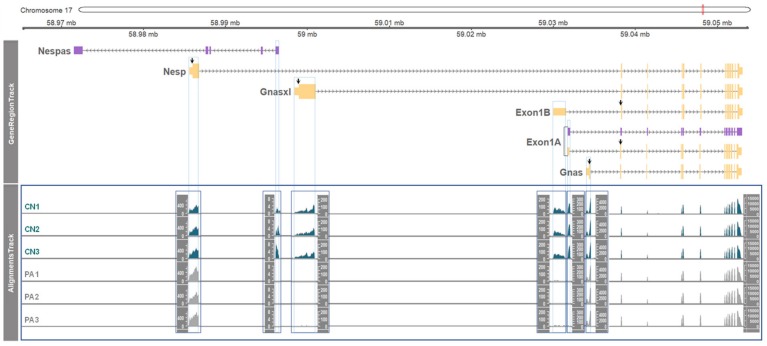
Allelic gene expression in unique 1st exons of six transcripts from the *GNAS* locus. In GeneRegionTrack, the non-overlapping 1st exons are surrounded by blue lines which are extended through AlignmentsTrack. The RNA-seq read coverages (or depths) extracted from BAM files are shown as counts (Y axis) in each AlignmentsTrack of CN (n = 3) and PA (n = 3). The track for shared exons 2–13 (toward the 3’ end) showed the highest coverage as indicated in the Y axis (far right). Due to the difference in the AlignmentsTrack height, separate AlignmentsTracks were generated and enclosed by rectangles.

To investigate the parental-origin effect on DNA methylation in the GNAS locus, methylation status between parthenotes (PA) and normal controls (CN) were compared. Using whole genome bisulfite sequencing (WGBS), DNA methylome was produced at single-base resolution. In the locus, major DMRs were grouped into three large clusters (Figure 1). In PA, hypermethylated regions in the vicinity of the promoter regions, or in the vicinity of the 1st exons, of Gnasxl, Nespas, Exon1B, and Exon1A, were identified (Figure 1; dotted blue boxes). Since CN has one paternal and one maternal allele and PA has two copies of maternal alleles, this hypermethylation in PA could be derived from the presence of an extra maternal allele. Especially, paternal allele-specific expression of Exon1B was first identified in the porcine species in this study (as shown below in Figure 2), and maternal methylation in the promoter region of Exon1B was identified (Figure 1). The corresponding transcript of Exon1B was absent in other mammals such as humans, mice, and cattle based on our PubMed, Ensembl, and UCSC database search. In addition, a large methylation “canyon” was shown in the vicinity of promoter regions of Nesp from the PA model, indicating unmethylated or hypomethylated CpGs in the two maternal alleles of PA (Figure 1; a dotted red box). Hypermethylation in the corresponding promoter region of Nesp in CN was detected and it indicates paternal methylation occcurred at this locus. All reported transcripts from this region in the NCBI Gene database (https://www.ncbi.nlm.nih.gov/gene) were grouped into transcripts depicted in Figure 1 based on their alternative promoter usages (Supplementary Figure S1), and Nespas (LOC106506734; GenBank assession number XR_001302255.1) was derived from the NCBI Nucleotide database (https://www.ncbi.nlm.nih.gov/nuccore).

### 3.2. Paternal or Maternal Gene Expression in the GNAS Locus

Next, expression levels of each transcript from the *GNAS* locus were examined by RNA-seq. To this end, the read coverage of unique exons (i.e., the non-overlapping first exons as displayed in Figure 1) from the six transcripts was compared. The height and coverage of bars displayed accumulated numbers of reads spanning the indicated exons of each transcript (Figure 2). As visualized, there were three patterns: paternal expression (as the expression occurred only in CN of *Nespas*, *Gnasxl*, *Exon1B*, and *Exon 1A*), maternal expression (as the level of expression in PA with two copies of maternal alleles was approximately two-fold higher in *Nesp* compared to CN with one maternal allele) and biallelical expression (as the level of expression in CN and PA was equivalent in *Gnas* and shared 2nd–13th exons). 

**Figure 3:**
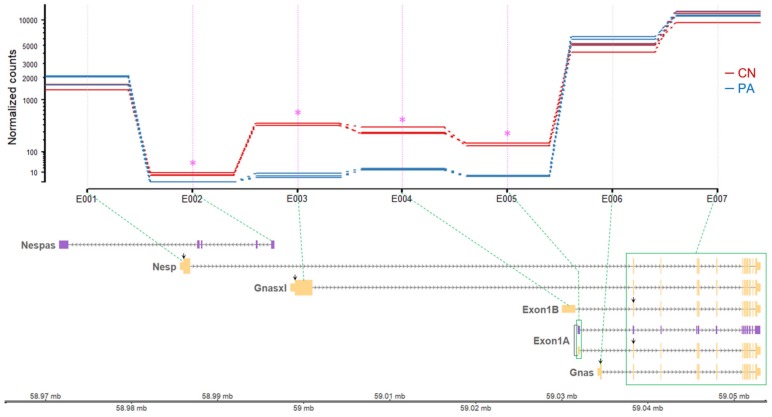
Differential usage of unique exons from the GNAS locus. The read counts from individual fetus samples were normalized by estimated size factors and presented on the log scale (Y axis). The size factors were estimated by relative sequencing depth. The exon counting bins (E00X; X axis) correspond to the 1st exons of six transcripts and merged shared exons 2 through 13 (a green box) from the GNAS locus. Each exon counting bin and its corresponding exon are linked by green dotted lines. Differential exon usage was tested by the function test for DEU in the DEXSeq package. Shown in pink stars are the exons that showed significant differential exon usage. CN, control; PA, parthenote.

To validate these expression patterns, differentially expressed exonic regions were screened using the DEXSeq package [18]. In the *GNAS* locus, four significant differential exonic regions were found which are corresponding to the 1st exons of *Nespas*, *Gnasxl*, *Exon1B*, and *Exon 1A* (Table 1 and Figure 3). All of those four exons showed significantly higher expression in CN, indicating paternal expression of the corresponding transcripts containing those four exons. In addition, the expression of the 1st exon of *Nesp* tended to increase in PA although it was statistically nonsignificant, suggesting higher expression of the corresponding *Nesp* transcript in maternal alleles (Table 1 and Figure 3). The exonic regions corresponding to the 1st exon of *Gnas* and other exonic regions toward the 3ʹ end did not show difference between CN and PA, indicating biallelic expression (Table 1). As shown in Figure 3, the expression of *Gnas* transcript in both CN and PA was substantially higher than other transcripts, as the number of normalized counts mapped to the 1st exon of *Gnas* was more than 3-fold higher compared to the 1st exon of *Nesp* and at least 48-fold higher on average than other 1st exons. 

### 3.3. DNA Methylation and Allele-Specific Expression in the Porcine GNAS Locus Showed a Conserved Pattern

**Figure 4:**
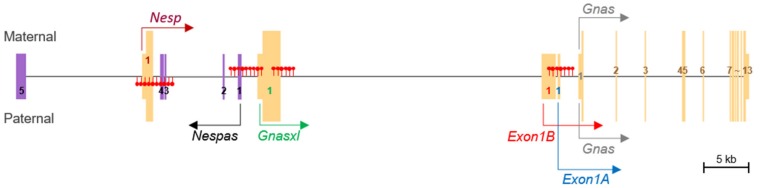
Schematic representation of methylation and expression patterns of the porcine *GNAS* locus. All transcripts were combined into a single merged transcript. Filled red lollipops denote either paternal or maternal methylation in the corresponding genomic regions. Maternal (*Nesp*), paternal (*Nespas*, *Gnasxl*, *Exon1B*, and *Exon 1A*), and biallelical (*Gnas*) expressions and the direction of transcription are represented. Exon numbers are displayed in unique 1st exons, exons from *Nespas* (toward the 5’ end), and shared exons (2nd–13th) between transcripts.

Overall, DNA methylation and maternal, paternal, and biallelical expression patterns of transcripts were conserved between humans and pigs. The three expression patterns, along with differentially methylated regions, were summarized and depicted (Figure 4). In addition, biallelical expression of the upstream *STX16* gene (approx. 120 kb upstream of *Nespas*), which contains a regulatory element for the *GNAS* locus in humans [19,20], was detected, as CN and PA expressed *STX16* equivalently (Supplementary Figure S2).

## 4. Discussion

Here we report a novel imprinted transcript, *Exon1B*, in the *GNAS* locus of the pigs through methylome and transcriptome analyses on parthenogenetic fetuses. In addition, our findings on parental-origin-specific methylation status and exon-based transcription patterns emphasized the adequacy of the animal model and subsequent evaluating strategies for imprinting in a genomic context. Therefore, subsequent studies may employ this approach to reveal parental origin-specific imprinting landscape, through examining expression of particular imprinted transcripts by unique exons when multiple overlapping exonic regions are present. For imprinting research, uniparental embryos and placenta having solely maternal or paternal genome have been generated using reproductive technologies and are useful models to discover the roles of imprinted genes [21,22]. Our generation of parthenogenetic porcine fetuses, combining whole genome bisulfite sequencing and RNA sequencing of each individual in control and parthenote groups, further decreased genetic variations that could affect gene expression patterns independent from genomic imprinting status. 

The *GNAS* locus produces various transcripts due mainly to alternative promoter usage in humans, mice, and pigs [23,24] (Supplementary Figure S1). Among those transcripts are five major transcripts (*Nesp*, *Gnasxl*, *Exon1B*, *Exon 1A*, and *Gnas*) and one antisense transcript (*Nespas*). Mutations and/or epigenetic changes in the *Gnas* transcript are implicated in pseudohypoparathyroidism (PHP) which is characterized by resistance to parathyroid hormone (PTH) and developmental abnormalities [25]. Noticeably, symptomatic differences are present between maternal and paternal mutations of *Gnas*, where maternal mutations are further related to obesity and neurocognitive defects [25]. On the other hand, regarding epigenetic state, the promoter and first exon of mouse *Gnas* were reported to be non-methylated on either paternal or maternal allele, indicating biallelical expression of the *Gnas* transcript [26]. Thus, the *Exon1A* germline DMR (gDMR) reported in the upstream region of *Gnas* transcript (approximately from positions −3400 to −939 relative to the *Gnas* transcription start site) on the maternal allele might not affect the *Gnas* transcript in most mouse tissues, leading to biallelical *Gnas* expression (reviewed in [27]). Exceptionally, in some tissues, allele-specific imprinting patterns of *Gnas* also exist (e.g., maternal allele-specific expression of Gsα in renal proximal tubules, white adipose tissue, brown adipose tissue, and a specific brain region such as the paraventricular nucleus of the hypothalamus (PVN)) [28,29]. It was further shown, in a tissue in which exceptional maternal allele-specific expression of *Gnas* occurs, that depletion of the *Exon1A* gDMR on a paternal allele gave rise to derepression of *Gnas* on a paternal allele and subsequent biallelical *Gnas* expression [30], indicating the additional role of *Exon1A* gDMR on the *Gnas* transcript in particular tissues besides its effect on the *Exon1A* transcript in most tissues. In our study on whole porcine fetuses, the *Exon1B*-*Exon1A* DMR located approximately from positions ‒3663 to ‒921 relative to the *Gnas* transcriptional start site (Figure 1). This 921-bp-gap between the *Exon1B*-*Exon1A* DMR and the porcine *Gnas* transcription start site and non-methylation of the promoter of *Gnas* which lies in the gap are probable causes of non-imprinting status of the *Gnas* transcript in porcine fetuses as shown in its biallelical expression (Figure 2 and Figure 3, and Table 1). 

Rather, maternal hypermethylation at this *Exon1B*-*Exon1A* DMR might affect the promoter and first exon of *Exon1A* and *Exon 1B* transcripts due to the proximity, and subsequent paternal expression of those transcripts was confirmed by transcriptome analysis in this study. Furthermore, consistent with previous mouse and human studies [24,26], the *Nespas*-*Gnasxl* gDMR and *Nesp* DMR were identified in the vicinity of the promoters and first exons of each of those transcripts. In mouse studies, depletion of the *Nespas*-*Gnasxl* gDMR on paternal allele led to deregulation of all transcription in the locus and a loss of *Nesp* DMR which is a secondary DMR that is normally established during early embryogenesis [31]. In our porcine study, the paternal expression patterns of *Nespas* and *Gnasxl* transcripts might be due to maternal hypermethylation at the *Nespas*-*Gnasxl* gDMR, and paternal hypermethylation at the *Nesp* DMR might further resulted in a tendency to maternal expression of the *Nesp* transcript. Of note, as mentioned in Introduction, opposing phenotypes derived from deficiency of the paternally expressed *Gnasxl* (leanness) and maternal depletion of *Gnas* (obesity) [5,6,7] imply essential roles of those transcripts in the regulation of metabolic states. In the pigs, their roles on the performance traits will need to be investigated while considering a substantial expression difference between *Gnasxl* and *Gnas* transcripts (Figure 3). *Nespas*, also named *Gnas-as1*, is a paternally expressed macro noncoding RNA (ncRNA) transcript (~1.5 kb) which runs antisense to *Nesp* [24]. Besides DNA methylation, long or macro ncRNAs also regulate imprinted gene clusters [32]. However, in our study, the absolute amount of *Nespas* transcript was very limited in the porcine fetuses, although its differential expression between PA and CN was detected (Table 1). In this sense, the paternal methylation in *Nesp* DMR, instead of paternal expression of *Nespas*, was probably the main cause of maternal, or paternally down-regulated, expression of *Nesp*. 

In addition, it has been reported that the biallelically expressed upstream *STX16* gene consists of *cis*-acting regulatory element for the *GNAS* locus, such that maternally inherited microdeletion in around exon 4 of the *STX16* gene led to a loss of methylation at the *Exon1A* DMR (approx. 220 kb downstream of the *STX16* exon 4) and biallelical *Exon1A* expression which are associated with autosomal-dominant PHP type Ib (AD-PHP1B) [19,20]. In the pigs, the distance between exon 4 of *STX16* and *Exon1B-Exon1A* DMR is shorter (approx. 175 kb), but according to our literature search there was no report on maternally inherited microdeletion in the porcine *STX16* gene that reduced methylation at *Exon1B-Exon1A* DMR. In our PA model (i.e., a model of maternal uniparental disomy, or UPD), bimaternal expression of *STX16*, did not affect the methylation status and expression of *Exon1A* as well as *Exon1B*. Furthermore, in comparison with other UPD-related imprinted loci that are significantly associated with genetic diseases [33,34], the overall effect of maternal UPD on the *GNAS* locus might not be substantial, because of biallelical expression of the predominant *Gnas* transcript and lower amounts of other parental-origin-specific transcripts (i.e., *Nesp*, *Nespas*, *Gnasxl*, *Exon1B*, and *Exon 1A*). One limitation of the current study was that the tissue- or region-specific imprinting status mentioned above could not be examined because whole fetuses were sampled for the analysis due to their small size.

Taken together, the parthenogenetic fetuses were used as a model to explore the imprinting status of the porcine *GNAS* locus. Our results indicated that the *GNAS* locus produces paternally expressed *Nespas*, *Gnasxl*, *Exon1B*, *Exon 1A*, maternally expressed *Nesp*, and biallelically expressed *Gnas* in the pig fetus. Our findings also include identification of three DMRs, which are ICRs of the *GNAS* locus: paternally methylated *Nesp* DMR, and maternally methylated *Nespas*-*Gnasxl* DMR and *Exon1B–Exon1A* DMR. Overall, this study provided an efficient strategy of identifying imprinted genes or transcripts for future studies. 

## Figures and Tables

**Table 1:** Analysis of differential exon usage (DEU) in the *GNAS* locus performed by DEXSeq.

ECB ^1^	Width ^2^	Exon	M.N.count ^3^ (CN)	M.N.count (PA)	log2fold ^4^ (PA/CN)	*p*-Value ^5^	Padjust ^6^
E001	1220	*Nesp* 1st exon	1473.00 ± 115.11 ^b^	2017.00 ± 176.12 ^b^	0.45	0.18	0.92
E002	350	*Nespas* l 1st exon	6.00 ± 1.53 ^a^	0.0033 ± 0.0033 ^a^	−10.81	1.59E − 06	7.32E − 04
E003	1972	*Gnasxl* 1st exon	402.00 ± 4.04 ^ab^	5.00 ± 2.08 ^a^	−6.33	0.00E + 00 ^7^	0.00E + 00
E004	1506	*Exon1B* 1st exon	298.00 ± 21.96 ^ab^	17.33 ± 1.86 ^a^	−4.10	3.74E − 82	2.07E − 77
E005	205	*Exon1A* 1st exon	155.67 ± 11.89 ^ab^	3.67 ± 0.67 ^a^	−5.41	6.63E − 84	4.88E − 79
E006	481	*Gnas* 1st exon	4621.00 ± 423.42 ^c^	6147.67 ± 536.13 ^c^	0.41	0.13	0.88
E007	14,754	Shared exons (2nd–13th) ^8^	10,757.33 ± 610.78 ^d^	12,251.00 ± 568.18 ^d^	0.19	0.81	1.00

^1^ Exon counting bin. ^2^ Width of exon counting bins in base pairs that correspond to exons listed in the 3rd column. ^3^ Mean of normalized count (M.N.count) which is presented as mean ± SEM. ^4^ Log 2 fold changes of averaged normalized counts that are mapped to exon counting bins for PA against CN. ^5^ p-value from the test for differential exon usage conducted by the function testForDEU in the DEXSeq package. ^6^ Benjamini–Hochberg (BH) adjusted *p*-value. ^7^ p-value of 0.00E + 00 represents that the significance was not measurable because it reached a maximum significance set by the program. ^8^ Shared exons 2 through 13 which were merged into one exon counting bin. Different alphabets a–d in M.N.count colums denote statistical significance at *p* < 0.05 in one way ANOVA followed by Tukey’s post hoc test.

## Supplementary Materials

The following are available online at https://www.mdpi.com/2073-4425/11/1/96/s1, Supplementary Figure S1: Alternative promoter usages and subsequently generated splicing isoforms in the porcine *GNAS* complex locus. Supplementary Figure S2: Transcriptome and methylome profiling of the upstream region of the *GNAS* complex locus. Supplementary Table S1: Whole genome bisulfite sequencing (WGBS) data containing methylation ratio. Supplementary Table S2: Differentially methylated region (DMR) called by the metilene software. Supplementary Table S3: Analysis of differential exon usage (DEU) by the DEXSeq program.
