# Supplementary material for: Identification of a Novel Imprinted Transcript in the Porcine GNAS Complex Locus Using Methylome and Transcriptome of Parthenogenetic Fetuses
Source: Genes (Basel). 2020 Jan 14;11(1):96. doi: 10.3390/genes11010096 (PMC7017182; doi:10.3390/genes11010096)
Supplement: Supplementary file 1 [file genes-11-00096-s001.zip › S.Figure1.pdf]

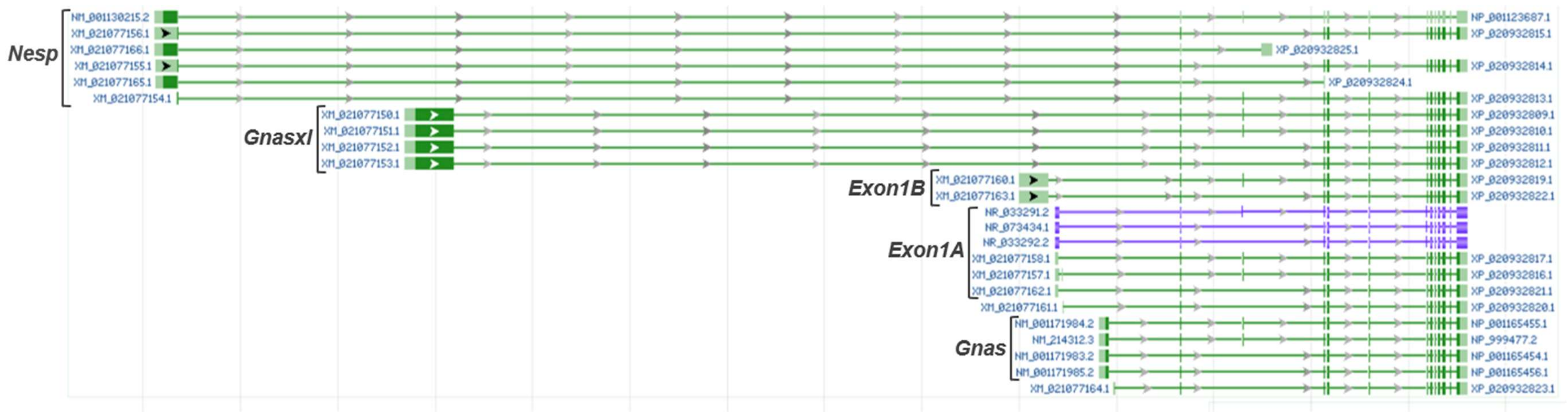


**Supplementary Figure 1.** Alternative promoter usages and subsequently generated splicing isoforms in the porcine *GNAS* complex locus. Protein coding (green-colored) and noncoding (purple-colored) transcripts originated from the *GNAS* complex locus are represented based on NCBI Gene database (https://www.ncbi.nlm.nih.gov/gene). Five major groups (*Nesp*, *Gnasxl*, *Exon1B*, *Exon 1A*, and *Gnas*) are indicated with brackets.
