# Supplementary material for: Identification of a Novel Imprinted Transcript in the Porcine GNAS Complex Locus Using Methylome and Transcriptome of Parthenogenetic Fetuses
Source: Genes (Basel). 2020 Jan 14;11(1):96. doi: 10.3390/genes11010096 (PMC7017182; doi:10.3390/genes11010096)
Supplement: Supplementary file 1 [file genes-11-00096-s001.zip › S.Figure2.pdf]

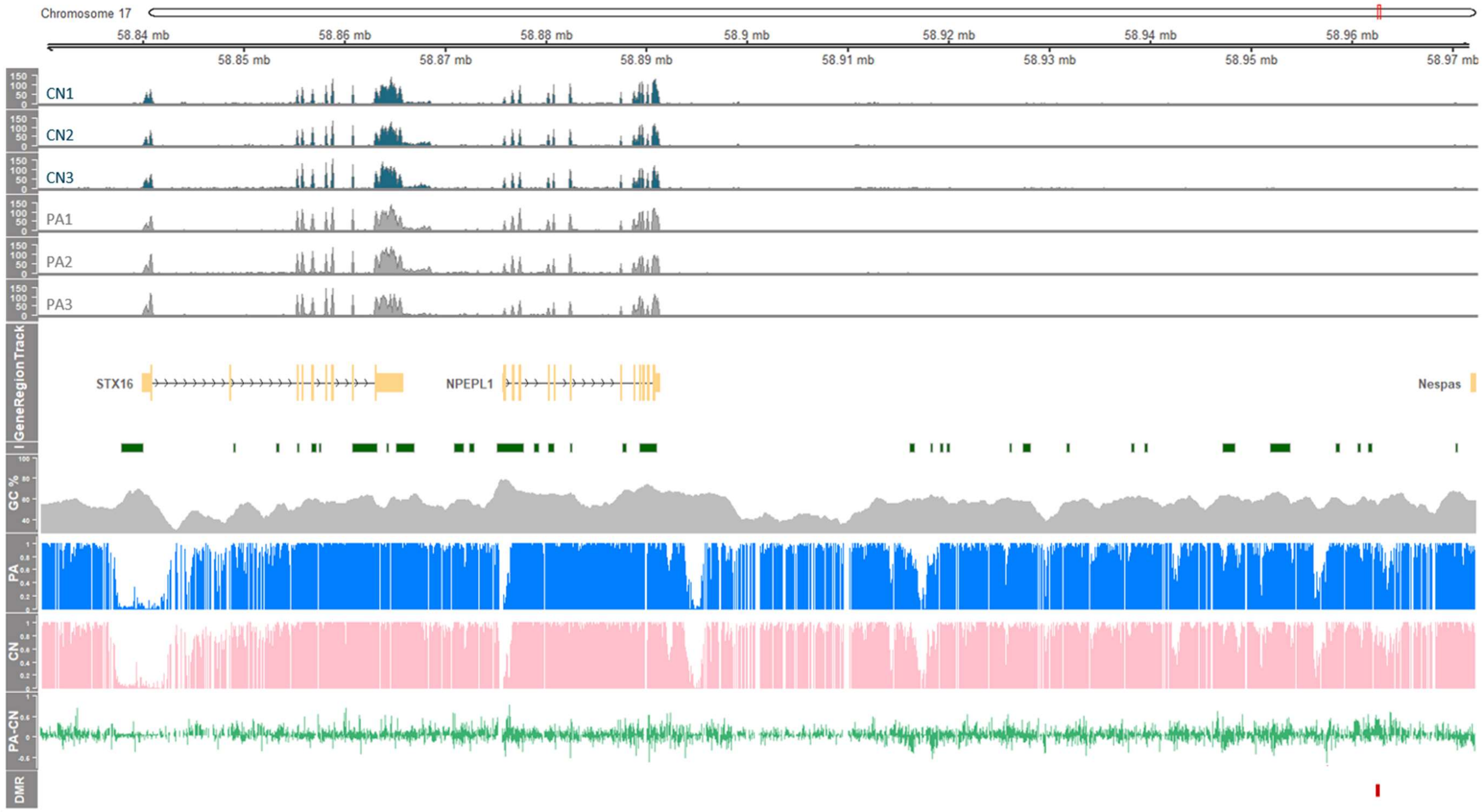

Supplementary Figure 2 Transcriptome and methylome profiling of the upstream region of the *GNAS* complex locus. The *STX16* and *NPEPL1* genes are depicted in the upstream of the *GNAS* complex locus. Biallelical enrichments of RNA-seq reads are displayed with a comparable expression level between CN (dark cyan) and PA (grey). Mean methylation ratios are shown in the bottom tracks representing PA (blue histogram lines) and CN (pink histogram lines). PA-CN track indicates the difference in mean methylation ratios between PA and AI. A significant DMR (FDR < 0.05) is marked by a red horizontal bar. *I*, CpG islands; *GC %*, GC content.
